# Combination therapy of cytoreductive surgery and hyperthermic intraperitoneal chemotherapy for recurrent leiomyomatosis peritonealis disseminata with endometriosis: A case report

**DOI:** 10.1016/j.heliyon.2023.e19794

**Published:** 2023-09-02

**Authors:** Xiaoli Xiao, Cong Wang, Yuyuan Zhang, Fang Li, Huan Zhang, Ruiqing Ma

**Affiliations:** a Department of Gynecology, Aerospace Center Hospital, Beijing, 100049, China; b Department of Pathology, Aerospace Center Hospital, Beijing, 100049, China; c Department of Imaging, Aerospace Center Hospital, Beijing, 100049, China; d Department of Myxoma, Aerospace Center Hospital, Beijing, 100049, China

**Keywords:** Cytoreductive surgery, Hyperthermic intraperitoneal chemotherapy, Leiomyomatosis peritonealis disseminate, Endometriosis, Peritonectomy

## Abstract

**Background:**

Leiomyomatosis peritonealis disseminata (LPD) is a non-metastatic, homologous, multicentric benign disorder characterized by small leiomyomas scattered over the peritoneum and omentum. It is a rare and benign disease with invasive potential. LPD mainly attacks women of childbearing age but has also been reported in post-menopausal women, men, and young children. Non-specific clinical and imaging findings of LPD lead to difficult diagnoses and treatment.

**Case presentation:**

This study reports the case of a patient with recurrent LPD with endometriosis after multiple myomectomies and hysterectomy, who presented recurrent abdominal pain with progressive exacerbation. Imaging examinations showed irregular shadows in the pelvic cavity and multiple nodular changes in the peritoneum, which were considered malignant lesions. A solid mass sized 10 cm × 9 cm × 10 cm in the inferior pelvis and nodules scattered over the surface of pelvic and abdominal organs and the peritoneum were detected during the surgery. The patient was treated with cytoreductive surgery (CRS), peritonectomy, ovarian ablation, and hyperthermic intraperitoneal chemotherapy (HIPEC). The surgery was challenging, and the intraoperative bleeding reached 900 ml. However, the patient recovered well and achieved a tumor-free survival of 13 months.

**Conclusions:**

It was concluded that a combination of CRS, ovarectomy, and HIPEC might be one of the therapeutic strategies for recurrent LPD.

## 1 Background

Leiomyomatosis peritonealis disseminata (LPD) was initially reported in 1952 [1] as a non-metastatic, homologous, multicentric benign disorder characterized by small leiomyomas scattered over the peritoneum and omentum. It is a benign disease with great invasive potential. LPD mainly affects women of childbearing age. Most LPD cases are asymptomatic and are occasionally diagnosed during surgery. In some cases, abdominal pain and distention may be present. The pathogenesis of LPD has not been fully elucidated, which is closely linked with hormone dependence, the metaplastic potential of stem cells within the peritoneal cavity, and iatrogenic responses. Surgery is the primary treatment of LPD, and the surgical scope should be individualized according to fertility requirements. This case report describes a woman of childbearing age with recurrent LPD after multiple surgeries and endometriosis. After cytoreductive surgery (CRS), peritonectomy, ovarian ablation, and hyperthermic intraperitoneal chemotherapy (HIPEC), the patient achieved a tumor-free survival of 13 months.

## 2 Case presentation

A 40-year-old woman, G4P1, was admitted in October 2021 due to pain in the right lower abdomen that had persisted for eight years. Additionally, she had been experiencing an aggravation of this pain, accompanied by a sensation of abdominal expansion, for the past month. The patient had a cesarean in 2009 and a laparoscopic myomectomy in 2012 for a uterine fibroid (5 cm) at A hospital. She experienced right lower abdominal pain from 2013. In 2015 and 2018, she had surgeries to remove uterine fibroids and ovarian cysts at B and C hospitals, followed by six cycles of GnRH-a therapy. In November 2018, she received a hysteroscopic levonorgestrel‐releasing intrauterine system at D Hospital and underwent an ultrasound-guided endometriomas puncture in 2019. In 2020, she had a total abdominal hysterectomy, right adnexectomy, left salpingectomy, left ovarian cyst removal, and intestinal adhesion lysis at D hospital. Pathology post-surgery revealed a pelvic nodule, leiomyoma, uterine and cervical adenomyoma, right accessory endometriosis, and left ovarian endometrioma. Following three cycles of postoperative GnRH-a therapy, an ultrasound re-examination revealed a 4 cm solid mass.

**Fig. 1:**
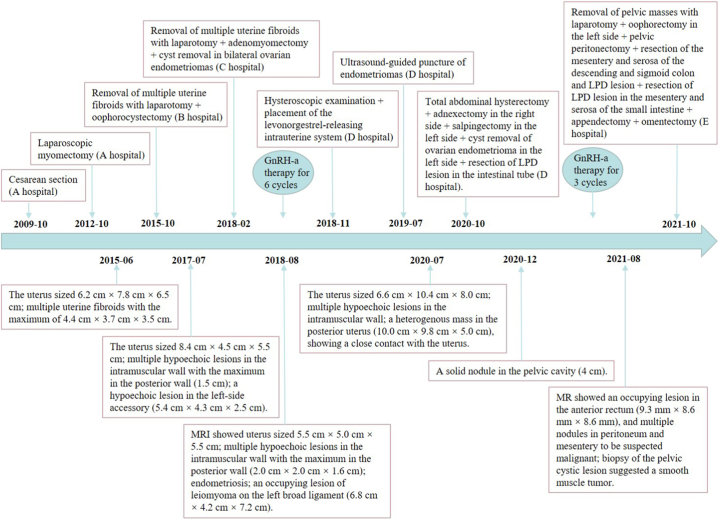
Timeline for the treatment.

However, the surgery did not alleviate the patient's right lower abdominal pain. As the pain gradually increased, oral painkillers became necessary for relief. In August 2021, a pelvic MRI and a contrast-enhanced MRI were conducted. They revealed a larger pelvic lesion (9.3 cm × 8.6 cm × 8.6 cm) than before, encroaching on the anterior rectum. Additionally, several nodules in the peritoneum and mesentery raised suspicions of malignancy (at E hospital). A subsequent biopsy of the pelvic cystic lesion suggested a smooth muscle tumor. However, the evidence for malignancy was inconclusive (at F hospital) (Fig. 1). The patient declined oral medication with highly effective progestogen, fearing thrombosis.

Exacerbating right lower abdominal pain, a heavy feeling, and sporadic back pain led the patient to seek help at the Aerospace Center Hospital. This pain lasted one to 7 h and could be alleviated with oral tramadol. Finally, it is noteworthy that the patient had a two-year history of smoking, consuming seven cigarettes daily. However, she quit smoking five years ago.

Physical examinations showed the patient in visible pain with a forced posture. The abdomen was soft and tender in the lower part, with no rebound tenderness. A pelvic examination revealed no hymen or obstruction in the vagina, and the previously sutured end remained smooth. A 10 cm mass was palpable in the pelvic cavity, fixed in the anterior rectum. It had a clear boundary, hard texture, significant tenderness, and limited mobility. The rectal mucosa was smooth, with another hard mass palpable 7 cm from the anus. This mass was mobile and without tenderness or bleeding upon digital rectal examination.

**Fig. 2:**
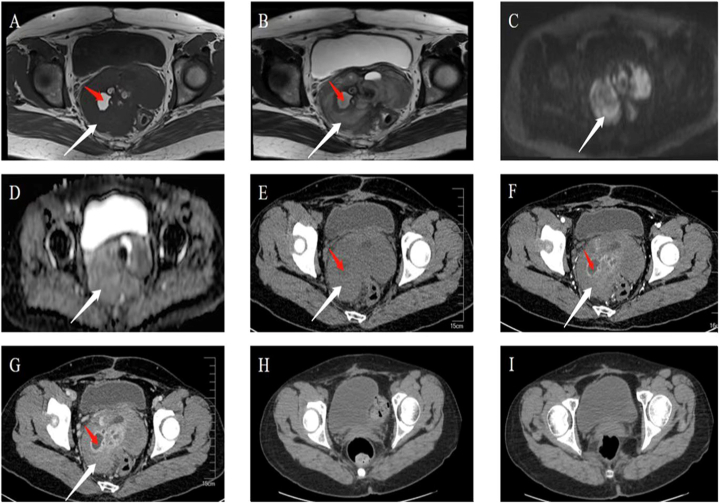
A-D. MRI scans showed multiple shadows of soft tissues (white arrow) with uneven signals and confluent intensity on T1WI (hypointensity, A), T2WI (mixed hyperintensity, B), DWI (hyperintensity, C), and ADC (hypointensity, D). The signal was uneven in the lesion, and multiple hyperintense lesions on T1WI and mixed hypointense lesions on T2WI were seen (red arrow). The lesion had a blurred margin with the adjacent rectum and sigmoid colon. E-G. Plain computed tomography (CT) (E), the CT arterial phase (F), and the venous phase (G) showed irregular lobular mass shadows behind the bladder (white arrow), in which the intensity was uneven and cystic regions were detectable (red arrow). The contrast-enhanced CT showed inhomogeneous enhancement in the lesion with local ring enhancement. Enhancement was not detectable in the cystic regions. The rectum and sigmoid colon were partially surrounded, and the boundary with the posterior wall of the bladder was blurred. Postoperative CT review: There was no abnormal density shadow in the pelvic cavity (H–I).

**Fig. 3:**
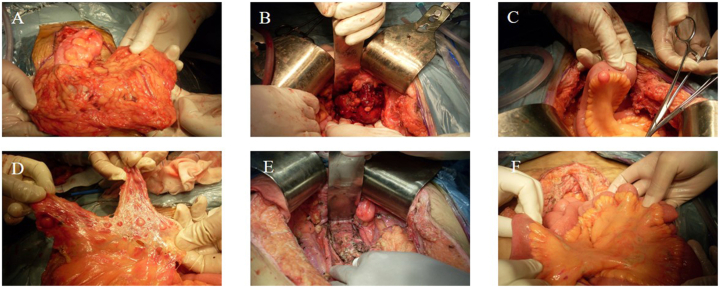
A-C. Surgical explorations of diffuse nodule implantations of the greater omentum (A), pelvic masses (B), and nodule implantations in the small intestinal mesentery (C). D. Isolation of mesentery and serosa of the small intestine. E. Postoperative pelvic cavity. F. Postoperative small intestinal mesentery.

**Fig. 4:**
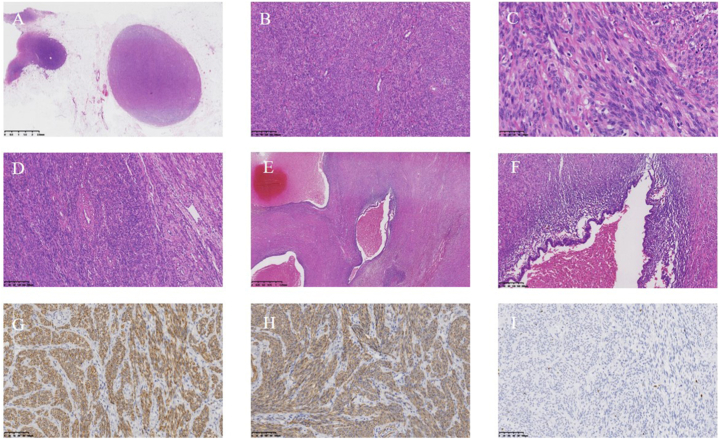
Pathological changes of the patient. A. H&E staining of multiple leiomyomas in the peritoneum of the right iliac fossa, with a clear boundary (magnification = 10 × ). B. H&E staining of braided spindle cells, scattered small muscular vessels, and cellular atypia and necrosis were not detectable (magnification = 100 × ). C. H&E staining of acidophilic spindle tumor cells with a blunt nucleus and enriched cytoplasm (magnification = 400 × ). D. H&E staining showed typical morphological characteristics of leiomyomas in the mesosigmoid and scattered thick and thin blood vessels (magnification = 100 × ). E. H&E staining showed cystic dilation and hemorrhage in the leiomyoma (magnification = 20 × ). F. H&E staining showed endometrial glands and stroma and hemosiderin deposition in the partial stroma (magnification = 100 × ). G, H, Immunohistochemical staining of smooth muscle markers (G) and desmin (I). Magnification = 200 × . I. Immunohistochemical staining of Ki-67, showing the Ki-67 index of 2% (magnification = 100 × ).

An MRI and CT scan of the pelvic area revealed an irregular mass and multiple nodules considered malignant (Fig. 2A–G). During surgery, a solid mass sized 1 cm × 0.9 cm x 1 cm was found in the lower pelvis. Many nodules were scattered over the pelvic and abdominal organs (Fig. 3A–D). The patient's CA125 level was 35.08 U/ml (see Fig. 4).

The exploratory laparotomy was performed in October 2021 at Aerospace Center Hospital. There were no detectable free ascites. Miliary nodules were present beneath the original incision site. The peritoneum of various areas had scattered miliary nodules ranging from 0.2 to 0.5 cm. Denser nodules of 0.5–1.5 cm were seen in the descending colon and related areas. Neoplastic adhesions sealed the pelvic cavity. During the operation, tissue was collected for pathology. Results hinted at a recurrent LPD spindle cell tumor. The patient then underwent CRS. Multiple resections were performed, including of the pelvic peritoneum, vaginal stump, and others. Full cytoreduction was achieved, denoted by a CC score 0 (Fig. 3E and F).

The patient had extensive abdominal nodules. Frozen section pathology indicated potential malignancy. Following family consultation, HIPEC treatment with 80 mg cisplatin in 4000 ml saline occurred. It was a challenging seven-hour surgery, with a blood loss of 900 ml. The patient was moved to intensive care afterward. Postoperative pathology showed multiple spindle cell tumors across various areas, with sizes from 0.5 cm × 0.5 cm × 0.5 cm–10 cm × 8 cm × 8 cm. Other findings included a pelvic tumor from endometriosis, a corpus luteum and corpus albicans in the left ovary, and chronic appendicitis. The fibrofatty tissues in the ligamentum teres hepatis and tumors were not detectable. Immunohistochemistry showed positive results for CD34, SMA, desmin, focal CD10, ER, PR, and 2% Ki-67, but negative for CD117, S-100, and Dog-1 (Fig. 4A–I).

Eleven days post-surgery, the patient was discharged. She was followed up for 13 months, showing tumor-free survival (Fig. 2H–I). While abdominal pain was gone, menopausal symptoms arose. Hormone tests showed luteinizing hormone at 44.87 IU/L, follicle-stimulating hormone at 108.8 IU/L, prolactin at 6.62 μg/L, progesterone at 0.16 ng/mL, estradiol less than 15 pg/ml, and testosterone at 12.08 ng/dL. The CA125 level fell to 4.0 U/ml.

## 3 Discussion and conclusions

LPD is a rare disease with invasive potential, but it is histologically benign. It mainly affects women of childbearing age, but it occasionally affects post-menopausal women [2,3], young children, and men [4]. Most LPD cases are asymptomatic and non-specific. As a result, the exact prevalence of LPD is unclear [5].

The pathogenesis of LPD has not been fully elucidated. LPD lesions shrank or regressed after GnRH-a therapy or treatment with aromatase inhibitors, further confirming the hormone dependence theory. In this case, postoperative positive estrogen and progesterone receptors and 13 months of tumor-free survival after ovarian ablation were consistent with the hormone dependence theory. However, short-term ovarian ablation with six cycles of GnRH-a after the fourth operation and three cycles of GnRH-a after the seventh unilateral oophorectomy failed to prevent the development of uterine fibroids, adenomyoma, and endometriosis. It could also not prevent the recurrence of LPD, which could not be explained by the hormone dependence theory. LPD also affects post-menopausal women, men, and infants, indicating the potential involvement of other theories.

Second, LPD may be caused by the metaplastic potential of stem cells within the peritoneal cavity [6]. The recurrent LPD, uterine fibroids, and endometriosis may originate from totipotent stem cells in the subcutaneous peritoneum, which is consistent with a previous case report involving seven LPD patients with endometriosis within the same lesions [7].

Third, the iatrogenic theory is involved in the pathogenesis of LPD. The patient was treated with an unindicated cesarean section followed by a laparoscopic myomectomy. According to the operation time and the hospital, unprotected intraabdominal morcellation was not applied. Immunohistochemical staining showed that SMA and desmin were strongly positive, suggesting that LPD shares partial common molecular and cytogenetic characteristics with those of uterine leiomyoma. It cannot be ruled out that the two surgeries jointly contributed to LPD and endometriosis, which accelerated the generation of totipotent stem cells in the subcutaneous peritoneum.

**Fig. 5:**
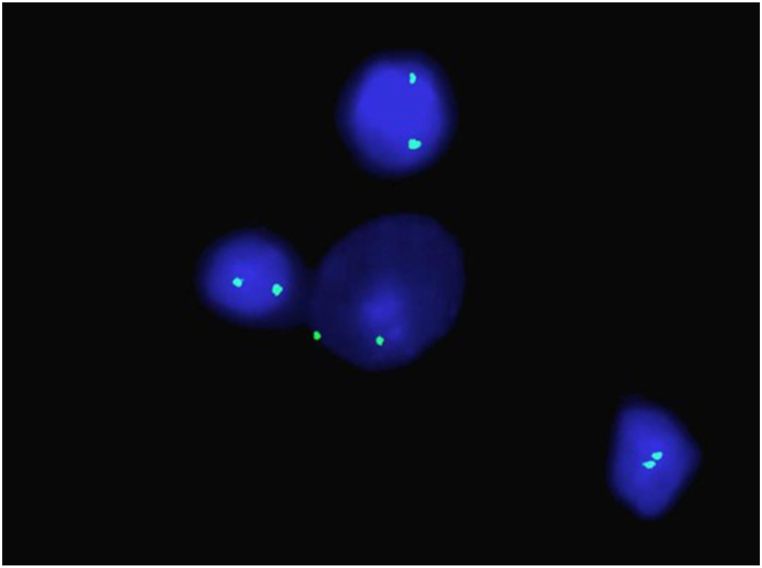
CEPX/CEPY FISH analysis; red and green spots indicated chromosomes Y and X, respectively, which revealed 100% XX signals (nuc ish (CEPX × 2) [500]).

Fourth, genetics theory is linked with the pathogenesis of LPD, and familial clustering of LPD has been reported [5]. Chromosomal abnormalities [8] or mutations in the *MED12* gene [9] are involved in the pathogenesis of LPD. This study performed karyotype analysis, and the data showed a 46, XX karyotype (Fig. 5). A history of uterine fibroids has not been reported within three generations for the patient. Whether smoking-induced gene mutations are the cause of LPD, in this case, remains unknown. Unfortunately, due to the high medical cost, the patient refused to be examined by genetic testing. It is believed that a single theory could not explain the cause of LPD due to rapid development, the short onset, and the progressive aggravation.

It is challenging to distinguish LPD from peritoneal implants of small malignancies based on preoperative imaging. Moreover, clinical manifestations of LPD are atypical and are mainly diagnosed through postoperative pathology. So far, global consensuses for treating LPD are scant [10]. In this case report, GnRH-a therapy was immediately given to the patient after total abdominal hysterectomy, adnexectomy on the right side, and resection of LPD lesions in the intestinal tube. However, LPD and endometrioma recurred in a short period, which may be attributed to the unresected peritoneum and omentum and the differentiation of the totipotent stem cells in the subcutaneous peritoneum into smooth muscle cells, endometrial glands, and mesenchyme.

Currently, CRS achieved by peritonectomy procedures and en-bloc resection of the viscera has been widely applied to treat tumors on the surface of the peritoneum, significantly reducing the recurrent rate and enhancing long-term survival. Multiple miliary nodules were examined in the patient's pelvic cavity, peritoneum, and omentum. The assessment of tumor burdens during surgical exploration is of great significance and ensures the feasibility of peritonectomy [11].

CRS is the most effective treatment for advanced epithelial ovarian malignancies, followed by intravenous paclitaxel and platinum chemotherapy. Systemic intravenous chemotherapy, however, is less effective in treating metastases due to poor peritoneal blood flow and less penetration of drugs into tumors. HIPEC has become a novel alternative that applies heated chemotherapy directly to abdominal tumors, causing cytotoxicity. HIPEC has emerged as an alternative to adjuvant or neoadjuvant therapy for advanced ovarian cancer and other malignant diseases with extensive peritoneal metastasis. In this case, we used HIPEC first in the world in benign-disseminated disorder because of potential malignancy indicated by intraoperative pathological diagnosis and fully informed consent from her family. Moreover, her clinical outcome has been great so far. The patient did not have HIPEC-induced adverse events, and the postoperative liver and kidney functions were normal. The use of HIPEC in benign-disseminated tumors needs cautious, and its effects are inconclusive.

During the surgery, malignant peritoneal implantation and metastasis could not be determined by the intraoperative frozen section pathology. After full communication with family members, HIPEC was actively performed to clear residual small tumors and free tumor cells in the pelvic cavity as much as possible, even though her CC score was zero. Heat is directly cytotoxic, enhances chemotherapeutic drug penetration into tumors, and synergizes with cisplatin to induce apoptosis in residual leiomyoma cells, which minimizes systemic adverse events. The patient did not have HIPEC-induced adverse events, and the postoperative liver and kidney functions were normal. The therapeutic effect of HIPEC in benign-disseminated tumors needs further exploration.

The study indicated the efficacy of CRS and ovarian ablation in recurrent LPD combined with endometriosis. This study reported, for the first time, the application of HIPEC to a patient with undetermined intraoperative pathology and extensive involvement of tumors in the abdominal cavity. This treatment may serve as a novel therapeutic strategy for recurrent LPD combined with endometriosis. The patient's quality of life after ovarian ablation deserves attention. Peri-menopausal symptoms and osteoporosis caused by low-level estrogen are the main adverse events after ovarian ablation in women of childbearing age. Moreover, low-level estrogen negatively influences bone metabolism, and the patient developed obvious pelvic pain after the surgery. Based on the add-back therapy for endometriosis after GnRH-a administration, the effect of low-dose estrogen supplements that improve the quality of life will be closely monitored [12]. In addition, considering the iatrogenic theory, the morcellation of uterine fibroids should be carried out with caution to avoid fragments remaining and prevent the development of LPD [13].

## Ethics approval and consent to participate

The study was reviewed and approved by the Ethics Committees of the Aerospace Center Hospital. Written consent was obtained from the patient.

## Consent for publication

Written informed consent was obtained from the patient to publish this case report and any accompanying images. A copy of written consent is available for review by the Editor-in-Chief of this journal.

## Funding

None.

## Author contribution statement

All authors listed have significantly contributed to the investigation, development and writing of this article.

## Data availability statement

Data will be made available on request.

## Declaration of competing interest

The authors declare that they have no known competing financial interests or personal relationships that could have appeared to influence the work reported in this paper.

## List of abbreviations

LPD: Leiomyomatosis peritonealis disseminata
CRS: After cytoreductive surgery
HIPEC: hyperthermic intraperitoneal chemotherapy
GnRH-a: Gonadotropin-releasing hormone agonist
MRI: magnetic resonance imaging
CT: computed tomography
CA125: cancer antigen 125
CC: completeness of cytoreduction
